# Topical Application of Siberian Pine Essential Oil Formulations Enhance Diabetic Wound Healing

**DOI:** 10.3390/pharmaceutics15102437

**Published:** 2023-10-09

**Authors:** Milica Nikolic, Marijana Andjic, Jovana Bradic, Aleksandar Kocovic, Marina Tomovic, Andjela Milojevic Samanovic, Vladimir Jakovljevic, Mirjana Veselinovic, Ivan Capo, Veljko Krstonosic, Nebojsa Kladar, Anica Petrovic

**Affiliations:** 1 Department of Pharmacy, Faculty of Medical Sciences, University of Kragujevac, 69 Svetozara Markovica St., 34000 Kragujevac, Serbia; bnikolic182@gmail.com (M.N.); andjicmarijana10@gmail.com (M.A.); jovanabradickg@gmail.com (J.B.); salekkg91@gmail.com (A.K.); marinapop@gmail.com (M.T.); 2 Center of Excellence for Redox Balance Research in Cardiovascular and Metabolic Disorders, 69 Svetozara Markovica St., 34000 Kragujevac, Serbia; andjela-kg@hotmail.com (A.M.S.); drvladakgbg@yahoo.com (V.J.); 3 Department of Dentistry, Faculty of Medical Sciences, University of Kragujevac, 69 Svetozara Markovica St., 34000 Kragujevac, Serbia; 4 Department of Physiology, Faculty of Medical Sciences, University of Kragujevac, 69 Svetozara Markovica St., 34000 Kragujevac, Serbia; 5 Department of Human Pathology, Sechenov First Moscow State Medical University, 8 Trubetskaya St., 119991 Moscow, Russia; 6 Department of Internal Medicine, Faculty of Medical Sciences, University of Kragujevac, 69 Svetozara Markovica St., 34000 Kragujevac, Serbia; miraveselinovic.m@gmail.com; 7 Center for Medical and Pharmaceutical Investigations and Quality Control, University of Novi Sad, 21000 Novi Sad, Serbia; ivan.capo@mf.uns.ac.rs (I.C.); nebojsa.kladar@mf.uns.ac.rs (N.K.); 8 Department of Histology and Embryology, Faculty of Medicine, University of Novi Sad, 21000 Novi Sad, Serbia; 9 Department of Pharmacy, Faculty of Medicine, University of Novi Sad, 21000 Novi Sad, Serbia; veljkokrst@yahoo.co.uk

**Keywords:** Siberian pine, topical application, wound repair, essential oil, oxidative stress, skin histology

## Abstract

This study aimed to develop novel topical formulations based on a natural component (0.5% of Siberian pine essential oil) and to assess its wound-healing capacity through macroscopic, histopathological, and biochemical examination. The phytochemical profile of *Pinus sibirica* essential oil (PSEO) and rheological analysis and safety potential of formulations were determined. The wound-healing effect was evaluated on an excision wound model in diabetic *Wistar albino* rats randomly divided into the following groups topically treated with (1) untreated, (2) 1% silver sulfadiazine, (3) ointment base, (4) gel base, (5) PSEO ointment, and (6) PSEO gel. Formulations containing PSEO were stable and safe for skin application. Three weeks of treatment with both PSEO formulations (ointment and gel) led to a significant reduction in wound size (98.14% and 96.28%, respectively) and a remarkably higher level of total hydroxyproline content (9.69 µg/mg and 7.26 µg/mg dry tissue, respectively) relative to the control group (65.97%; 1.81 µg/mg dry tissue). These findings were in correlation with histopathological results. Topically applied PSEO formulations were associated with a significant reduction in most of the measured pro-oxidants and enhanced activity of the antioxidant defense system enzymes (*p* < 0.05). Our findings showed that gel and ointment with PSEO demonstrated significant wound-repairing capabilities in the excision wound model.

## 1. Introduction

Skin, as a multifunctional organ, can be affected by chemical, physical, or mechanical agents, causing damage called wounds [1]. The importance of wounds is reflected in more patients suffering from acute or chronic wounds. Evidence suggests that chronic wounds impact the quality of life of nearly 15% of Medicare beneficiaries (8.2 million worldwide) with the annual cost of USD 28 billion, thus representing a large and growing social and economic burden that needs to be addressed urgently [2]. Chronic wounds involve diabetic ulcers, pressure ulcers, venous ulcers, arterial insufficiency ulcers, etc. However, diabetic wounds stand out as one of the most frequent (19–34% of diabetic patients suffer from wounds) and are associated with serious complications and, consequently, lethal outcomes [3].

The healing of the wound represents the restoration of injured tissue, and it is divided into four overlapping phases: (1) hemostasis begins with platelet aggregation along with vasoconstriction; (2) inflammation leads to the release of proinflammatory cytokines, growth factors, and small regulatory proteins; (3) proliferation involves epithelialization, an angiogenesis process, and the production of collagen; and (4) maturation starts with collagen remodeling and the formation of granulation tissue [1,4]. All the above-mentioned processes include numerous biochemical and cellular events which play an important role in wound healing. Although skin has inborn ability to regenerate, the healing process can be postponed or slowed by numerous pathological systemic changes characteristic of aging, inappropriate treatment, or diabetes mellitus (DM) [5]. DM represents a chronic metabolic disorder associated with serious microvascular and macrovascular complications that significantly affect patients’ economic status and life quality [6,7,8]. A major concern arises from the fact that wound healing is compromised in diabetic patients due to the interplay of various factors, including enhanced blood glucose level, hypoxia, oxidative stress pronounced inflammation, impaired microcirculation, and neuropathy. Importantly, diabetic wounds are prone to becoming infected with the risk of spreading infection on soft tissues, thus elevating the risk of developing serious complications that require medical care, hospitalization, and lower extremities amputation [9,10].

Current therapeutic approaches in wound-healing management are based on conventional oral and topical drugs containing anti-inflammatory or antimicrobial agents with numerous side effects (irritation and allergies) and microbial resistance, which may lead to prolonged treatment duration, decreased effectiveness, increased hospital stays, and medical costs [11]. Therefore, there is an urgent need to discover novel therapeutic strategies to improve patient’s daily activities and reduce morbidity. In addition to the already available synthetic drugs for wound management, a large number of medicinal plants and their products showed better wound healing profiles, lower toxicity, and affordable prices. New, plant-oriented strategies in wound treatment demonstrated magnificent outcomes by increasing wound contraction and decreasing inflammation [12,13].

*Pinus sibirica,* known as Siberian pine, is a coniferous tree belonging to the *Pinaceae* family. In traditional medicine, various parts of this plant were used for treating numerous diseases such as arthritis, rheumatism, and liver and kidney disorders due to its antioxidant, anti-inflammatory, antifungal, and antibacterial properties [14,15,16]. Nowadays, this plant plays a great role in the perfume, aromatherapy, and cosmetic industry [17]. Phytochemical studies of *P. sibirica* oil essential oil (PSEO) revealed that the most abundant compounds are monoterpenes (α-pinene, β-phellandrene, and δ-3-caren) and sesquiterpenes (β-bisabolene, β-humulene, and γ-muurolene) [14,15,16,17,18]. The above-mentioned monoterpenes applied alone showed extraordinary effects on wound healing by accelerating wound closure and increasing collagen deposition [19]. 

Searching the literature data, we revealed that there are no investigations that describe the wound-healing activity of PSEO. To address the aforementioned issues, our investigation was designed to formulate and characterize PSEO-based different topical semi-solid dosage forms (ointment and gel) as well as to evaluate wound-healing properties by performing a biochemical and histopathological analysis in diabetic animal models. 

## 2. Materials and Methods

### 2.1. Pinus Sibrica Essential Oil Obtaining

The essential oil of the Siberian pine (CAS number: 84012-35-1), obtained by hydro distillation from fresh needles of the Siberian pine, was purchased from “Alekpharm”, Belgrade, Serbia. This commercial essential oil is 100% pure and held in closed vials at 4 °C before use.

### 2.2. Identification of Pinus Sibirica Essential Oil Chemical Composition

To identify the qualitative and quantitative composition of the purchased *Pinus sibrica* essential oil, analysis was performed on an Agilent 6890B GC-FID instrument coupled to Agilent 5977 MSD using an HP-5MS capillary column (30 m × 0.25 mm; film thickness 0.25 µm) [20]. 

### 2.3. Composition and Preparation of Semi-Solid Samples 

PSEO was formulated into a topical ointment (0.5%) and topical gel (0.5%), shown in Table 1 and Table 2, respectively. The ointment base was made by precisely measuring and mixing components (cholesterol, lanolin, paraffinum liquidum, and vaselinum album). Afterward, the levigation method incorporated the *P. Sibirica* essential oil in the above-mentioned ointment base [21]. On the other hand, the gel base was prepared with carbomer, which was mixed with water. The produced dispersion was stirred with propylene glycol, and the gel was formed by adding a triethanolamine solution. The gel was preserved with sodium benzoate. In the prepared gel base, 0.5 g of PSEO was incorporated.

### 2.4. Rheological Characterization of Semi-Solid Formulations

Rheological properties of the gels and ointments were determined with a controlled-stress HAAKE Mars Rheometer (Thermo Scientific, Karlsruhe, Germany) at a constant temperature of 25 ± 0.1 °C, using serrated plate–plate P35/Ti/SE measuring geometry (the gap between plates was 1.000 mm). In continuous hysteresis loop tests, the shear rate continually increased from 0.001 to 100 s^−1^ during 120 s. After this, the shear rate was constant at 100 s^−1^ for 60 s, and finally, it decreased to 0 s^−1^ for 120 s. 

### 2.5. In Vivo Animal Experiments 

#### 2.5.1. Ethics Statement

The first part of the investigation was performed in the Laboratory for Pharmaceutical Technology. The second part of the study involving animals was conducted in the Laboratory for Cardiovascular Physiology of the Faculty of Medical Sciences, University of Kragujevac, Serbia, according to the European Directive for Protection of the Vertebrate Animals used for Experimental and Other Scientific Purposes 86/609/EES and the principles of Good Laboratory Practice. The experimental protocol was approved by the Ethics Committee of the Faculty of Medical Sciences, University of Kragujevac, Kragujevac, Serbia, number 01–12408.

#### 2.5.2. Acute Dermal Irritation Test 

A skin tolerance test was performed on healthy *Wistar albino* male rats (b.w. 200–250 g) according to the Organization for Economic Cooperation and Development (OECD) guidelines 404 [22]. The day before the examination, the fur was removed by closely clipping the dorsal area, and the test formulations were applied to a small area of the skin and covered with a gauze patch. Animals were divided into the following groups:

1. Animals treated with 0.5% PSEO ointment (*n* = 3);
2. Animals treated with 0.5% PSEO gel (*n* = 3);
3. Animals treated with the ointment base (*n* = 3);
4. Animals treated with the gel base (*n* = 3).

A total of 500 mg of tested samples were applied to the test site. Signs of morbidity, toxicity, or adverse skin reaction (edema, erythema, pruritus, and slight eschar formation) were examined in the first 4 h and for a period of 14 days [23,24].

#### 2.5.3. Wound-Healing Activity

##### Animals

For the excision wound-healing model, *Wistar albino* rats (250–300 g) were used and housed in clean cages under adjusted conditions (12:12 h light–dark cycle, at a temperature of 22 ± 2 °C, ad libitum access to water and food). 

##### Diabetes Mellitus Induction

All experimental overnight fasted rats (*n* = 48) received a single intraperitoneal injection of streptozotocin (STZ, 50 mg/kg) dissolved in a 0.05 M citrate buffer solution (pH = 4.5). After 72 h of STZ administration and 12 h fasting, the level of blood glucose was measured using a portable glucometer (Accu-Check^®^). Only rats with blood glucose levels > 11.1 mmol/L were selected for further experiments [25].

##### Excision Wound Model

Seven days after confirmation of DM, rats were anesthetized (i.p. injection of a mixture of xylazine (10 mg/kg) and ketamine (5 mg/kg)) in order to create excision wounds. Precisely, the backs of animals were shaved, cleaned with ethyl alcohol (70%), and created excision wounds (2 × 2 cm sized, 2 mm depth) with scissors and a scalpel [26]. 

After the procedure of wound excision was finished, animals were photographed, placed in individual cages, and divided into the following groups (*n* = 8 per group):

1. Animals without treatment (control group (CTRL));
2. Animas treated with a commercially available ointment 1% silver sulfadiazine (SSD group);
3. Animals treated with an ointment base (OINT group);
4. Animals treated with a gel base (GEL group);
5. Animals treated with a 0.5% *Pinus sibirica* essential oil ointment (PSOINT group);
6. Animals treated with a 0.5% *Pinus sibirica* essential oil gel (PSGEL group).

All the above-mentioned formulations were applied to created wounds with sterile cotton (0.5 g per day for three weeks) [12]. Afterward, rats were injected with a mixture of ketamine (10 mg/kg) and xylazine (5 mg/kg) in order to become anesthetized and sacrificed. Wound tissues were collected for histopathological analysis and determination of hydroxyproline content, while blood was taken to estimate the systemic redox status.

##### Wound Contraction

The wounds were photographed at the beginning of the experiment, after one week, two weeks, and three weeks of wound creation. Changes in wound area were measured using Image J software (version 2.9.0/1.54d), and the rate of wound contraction is presented as a percentage calculated by the formula below [27].

%Wound contraction = (Healed area*/Initrial wound area) × 100

*Healed area = Initial wound area − wound area on specific day

##### Hydroxyproline Content Determination

The wound tissue samples derived from experimental rats were dried at 60 °C during 12–18 h, measured to obtain similar weight, and hydrolyzed with 6 M HCl (1:10, *w*:*v*) at 130 °C for 4 h. After centrifugation (15 min at 3000 rmp) of hydrolyzed tissue samples, the supernatant was mixed with chloramine T. Twenty minutes later, an Ehrlich reagent was added to the prepared mixture at 60 °C for 30 min. Absorbance was scanned at 557 nm spectrophotometrically (Shimadzu UV-1800, Kyoto, Japan). A calibration standard curve was obtained from which hydroxyproline content was presented as μg/mg of dry tissue weight [28].

##### Systemic Redox State Determination

The parameters of the systemic redox state were measured from jugular vein blood samples collected at the moment of animal sacrifice according to our previously described protocol [12]. After centrifugation of blood samples, the supernatant was used for the determination of concentration oxidative stress parameters: nitrites (NO_2_^−^), index of lipid peroxidation (measured as TBARS), hydrogen peroxide (H_2_O_2_), and superoxide anion radical (O_2_^−^). Additionally, we collected the lysate of erythrocyte, which was used for obtaining parameters of the antioxidative defense system: level of reduced glutathione (GSH), the activity of antioxidant enzymes such as catalase (CAT), and superoxide dismutase (SOD).

##### Histology Analysis of Wound Tissue

Collected skin sections were fixed at a 10% buffered formalin solution at 4 °C for 24 h, dehydrated in isopropyl alcohol (70%, 80%, 96%, and 100%), embedded into the paraffin, and cut on a thickness of 5 μm using a rotating microtome (Leica, Wetzlar, Germany). All slides were stained with hematoxylin-eosin (H&E) and the following immunohistochemical markers: rabbit anti-MMP9 in a 1:25 dilution (Lab Vision; Thermo Scientific, Rockford, IL, USA); rabbit anti-collagen I in a 1:300 dilution (Abcam; Cambridge, UK); rabbit anti-CD34 in a 1:3500 dilution (Abcam; Cambridge, UK); and rabbit anti-Iba1 in a 1:8000 dilution (Abcam; Cambridge, UK). For visualization, we used Mouse and Rabbit EnVision Detection Systems, Peroxidase/DAB detection IHC kit (DAKO Agilent; Manchester, UK). Before the antibody application, we performed a retrieval reaction. For anti-collagen I and anti-MMP9, the TRIS base buffer (pH 8.2) was used, and for anti-CD34 and anti-Iba1, the citrate buffer (pH 6.0) was used. Antibodies were applied for 60 min at room temperature with Mayer’s hematoxylin counterstain and finally mounted with a DPX medium (Sigma–Aldrich, Darmstadt, Germany). An analysis of slides was performed under a Leica DMLB 100T professional biological microscope (Leica, Germany), and slides were scanned using a digital microscope VisionTek^®^ (Sakura, Osaka, Japan).

### 2.6. Statistic Analysis 

All presented data are shown as mean ± standard deviation (SD). Figures were made in Microsoft Excel. Data were assessed via a one-way ANOVA followed by Tukey’s multiple comparison post hoc tests, using IBM-SPSS, version 20. *p*-values less than 0.05 were considered statistically significant.

## 3. Results

### 3.1. The Essential Oil Chemical Composition

The chemical composition of PSEO, extracted from needles and identified via GC-MS, is presented in Table 3. Sixteen components were identified for PSEO, representing 99.32% of the total oil composition. Monoterpene hydrocarbons constituted half of the oil composition, with a total relative content of 88.62%. The most abundant compounds detected in this essential oil were α-pinene (40.19%), β-pinene (19.64%), and δ-3-carene (16.14%). 

### 3.2. Rheological Properties of Formulations

**Figure 1:**
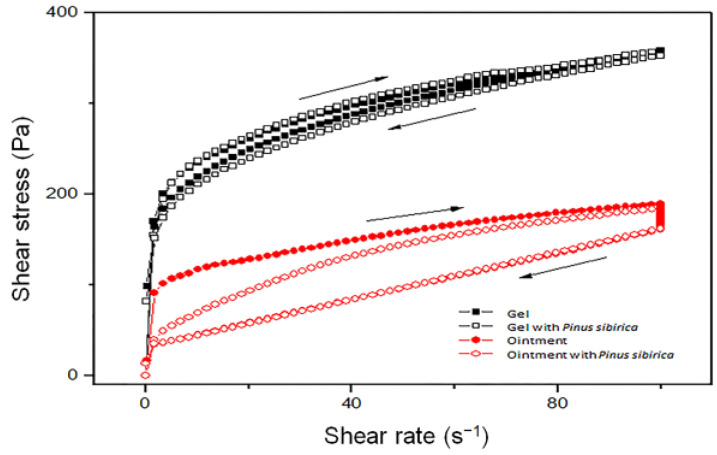
The dependence of shear stress on shear rate for gel and ointment with and without the addition of *Pinus sibirica* essential oil.

The flow curves of gels and ointments (with and without the addition of PSEO in formulation) are shown in Figure 1. It can be concluded that all formulations exhibited certain hysteresis loop areas in the plot of shear stress versus shear rate, which represents time-dependent, thixotropic flow behavior. This type of flow behavior is characteristic of gels and ointments and represents a desired property of topical preparations. The internal structure of systems breaks down under shearing and creates a hysteresis area. At the same time, the addition of PSEO in the formulation of gel or ointment did not produce a significant difference in the flow behavior, i.e., the flow curves almost overlapped.

### 3.3. Acute Dermal Irritation Test 

Based on our current knowledge, this is the first study investigating acute dermal irritation of Pinus sibirica ointment and gel. During the experiment, animals exposed to both PSEO ointment and PSEO gel showed no adverse reactions (edema, erythema, pruritis, or inflammation) or clinical signs of dermal toxicity. Additionally, there were no noticeable behavioral changes during 14 days. 

### 3.4. Wound-Healing Activity

#### 3.4.1. Wound Contraction

**Figure 2:**
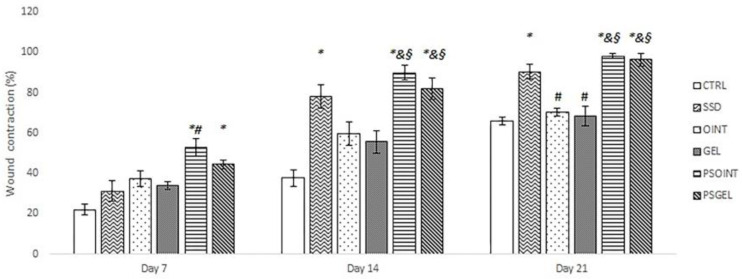
Influence of applied formulations on the wound contraction in diabetic rats. Values are mean ± standard deviation. * *p*  <  0.05 compared to CTRL group; ^#^ *p*  <  0.05 compared to SSD group; ^&^ *p*  <  0.05 compared to OINT group; ^§^ *p*  <  0.05 compared to GEL group; CTRL-untreated control group; SSD-1% silver sulfadiazine group; OINT-ointment base group; GEL-gel base group; PSOINT-*P.sibirica* essential oil ointment group; PSGEL-*P. sibirica* essential oil gel group.

**Figure 3:**
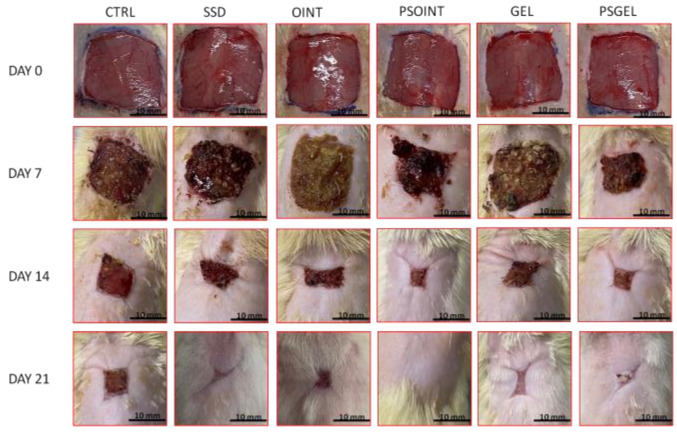
Impact of *P. sibirica* essential oil-based formulations on wound recovery during three weeks of application on the excision wound model (days: 0, 7, 14, and 21). CTRL-untreated control group; SSD-1% silver sulfadiazine group; OINT-ointment base group; GEL-gel base group; PSOINT-*P. sibirica* essential oil ointment group; PSGEL-*P. sibirica* essential oil gel group.

The progress of wound-healing contraction, examined over the three weeks, triggered via the topical application of PSEO ointment, PSEO gel, simple vehicle (ointment and gel), and 1% SSD, is shown in Figure 2 and Figure 3. After one week of application of PSEO formulations (both ointment and gel), we observed a significant reduction of wound area compared with the untreated group (CTRL group) (*p* < 0.05). Interestingly, the PSEO ointment-treated group manifested significantly higher wound-healing contraction relative to the SSD group (*p* < 0.05). As shown in Figure 2, after two weeks of topical application, the highest percent of wound contraction was noticed in groups treated with PSEO ointment and PSEO gel (percent of contraction: 89.86% and 81.84%, respectively). In fact, both formulations with PESO showed significantly higher wound contraction in relation to vehicle groups (*p* < 0.05) and the SSD group (*p* > 0.05), but the difference was not statistically significant. After three weeks of topical application, the maximum rate of wound contraction was observed in the PSEO ointment, PSEO gel group, and SSD group (98.14%, 96.28%, and 90.38%, respectively). The animals treated with both 0.5% PSEO ointment and 0.5% PSEO gel showed a significant increase in wound contraction compared to CTRL, OINT, and GEL groups (*p* < 0.05). 

#### 3.4.2. Determination of Hydroxyproline Content

**Figure 4:**
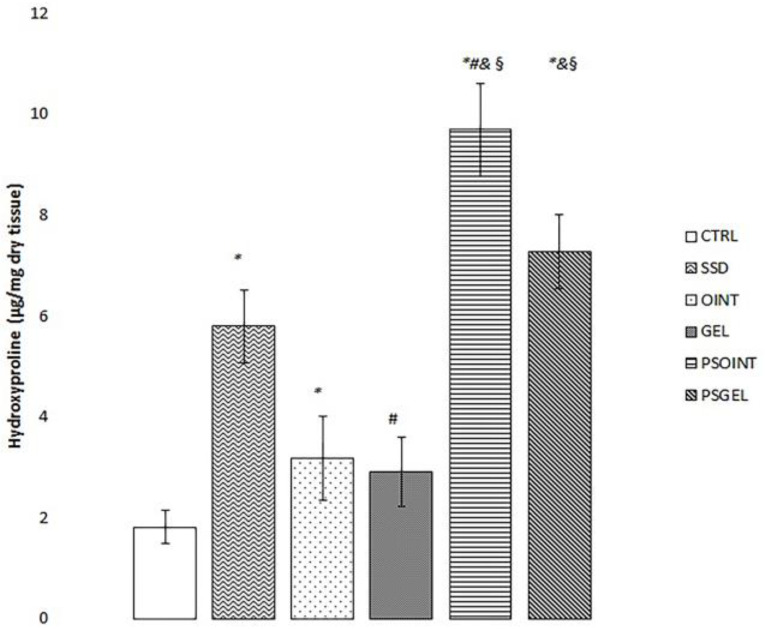
Hydroxyproline content after three weeks of PSEO topical formulations. The results are presented as the mean ± standard deviation. * Statistical significance at *p* < 0.05 in relation to CTRL group; *^#^* statistical significance at *p* < 0.05 in relation to SSD group; ^&^ statistical significance at *p* < 0.05 in relation to OINT group; ^§^ statistical significance at *p* < 0.05 in relation to GEL group.

The highest level of hydroxyproline was observed in the group treated with PSEO ointment for a period of three weeks (PSOINT group). Furthermore, we noticed significantly elevated hydroxyproline content of granulation tissue in both PSEO ointment and PSEO gel groups relative to the untreated group (CTRL) and groups treated with vehicles (OINT and GEL group). Moreover, a moderate effect on hydroxyproline level was determined in the SSD group compared to CTRL (untreated group) and the group treated with gel base only. Results are presented in Figure 4.

#### 3.4.3. Systemic Redox State Markers

**Figure 5:**
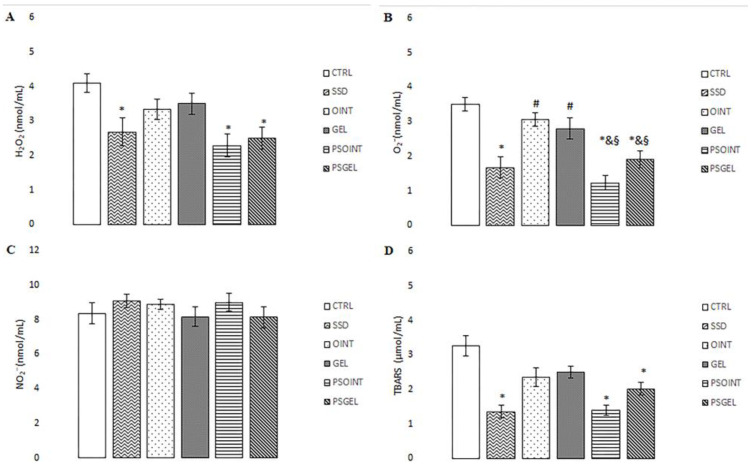
Impact of PSEO formulations on the pro-oxidative markers after three weeks of application: (**A**) H_2_O_2_^−^; (**B**) O_2_^−^; (**C**) NO_2_^−^; (**D**) TBARS. The results are presented as the mean ± standard deviation. * Statistical significance at *p* < 0.05 in relation to CTRL group; *^#^* statistical significance at *p* < 0.05 in relation to SSD group; ^&^ statistical significance at *p* < 0.05 in relation to OINT group; ^§^ statistical significance at *p* < 0.05 in relation to GEL group.

Levels of hydrogen peroxide (H_2_O_2_) release and TBARS (thiobarbituric acid-reactive substances) were remarkably decreased in groups treated with 1% SSD, 0.5% PSOINT, and 0.5% PSGEL relative to the untreated (CTRL) group. Additionally, topical treatment with *P. sibirica* essential oil (both ointment and gel) significantly diminished the level of superoxide anion radical (O_2_^−^) in comparison to control and vehicle groups. On the other hand, none of the topical treatments affected level nitrites (NO_2_^−^). All results are illustrated in Figure 5A–D.

**Figure 6:**
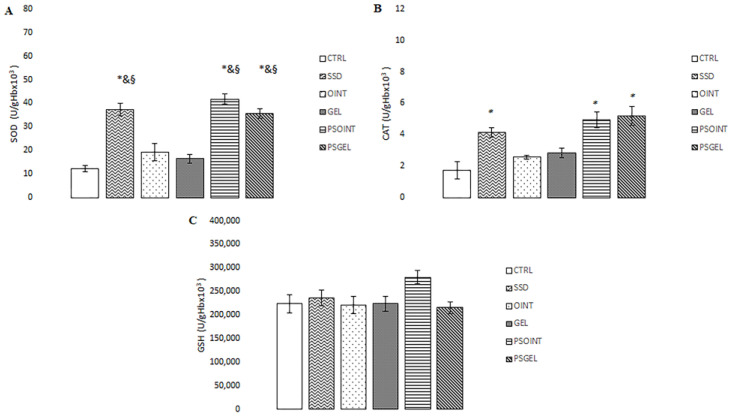
Effect of PSEO formulations after three weeks of application on antioxidative defense system parameters: (**A**) CAT; (**B**) SOD; (**C**) GSH. The results are presented as the mean ± standard deviation. * Statistical significance at *p* < 0.05 in relation to CTRL group; ^&^ statistical significance at *p* < 0.05 in relation to OINT group; ^§^ statistical significance at *p* < 0.05 in relation to GEL group.

Topical treatment with PSEO formulations (ointment and gel) and SSD significantly increased the activity of antioxidant enzymes (CAT and SOD) relative to the control group. Additionally, the activity of SOD was remarkably increased in PSOINT and PSGEL groups, comparing the values of this enzyme in groups treated with vehicle only (OINT and GEL group). Neither treatment made a difference in the level of GSH. Results regarding the activities of catalase (CAT), superoxide dismutase (SOD), and the level of reduced glutathione (GSH) are presented in Figure 6A–C. 

#### 3.4.4. Histopathological Analysis

**Figure 7:**
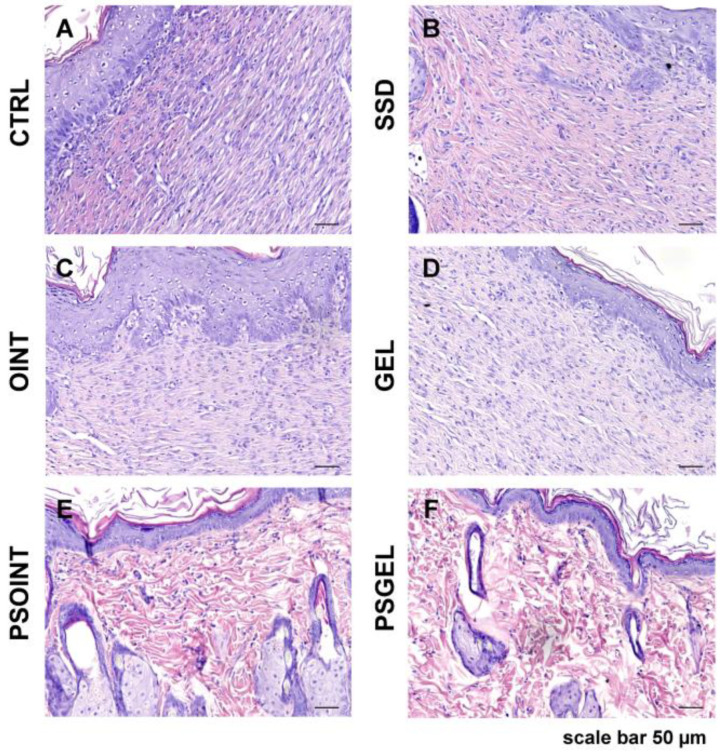
Histological changes in the skin wound section stained with hematoxylin-eosin (H&E). Scale bar, 50 μm. CTRL-untreated control group (**A**); SSD-1% silver sulfadiazine group (**B**); OINT-ointment base group (**C**); GEL-gel base group (**D**); PSOINT-*P. sibrica* essential oil ointment group (**E**); PSGEL-*P. sibirica* essential oil gel group (**F**).

**Figure 8:**
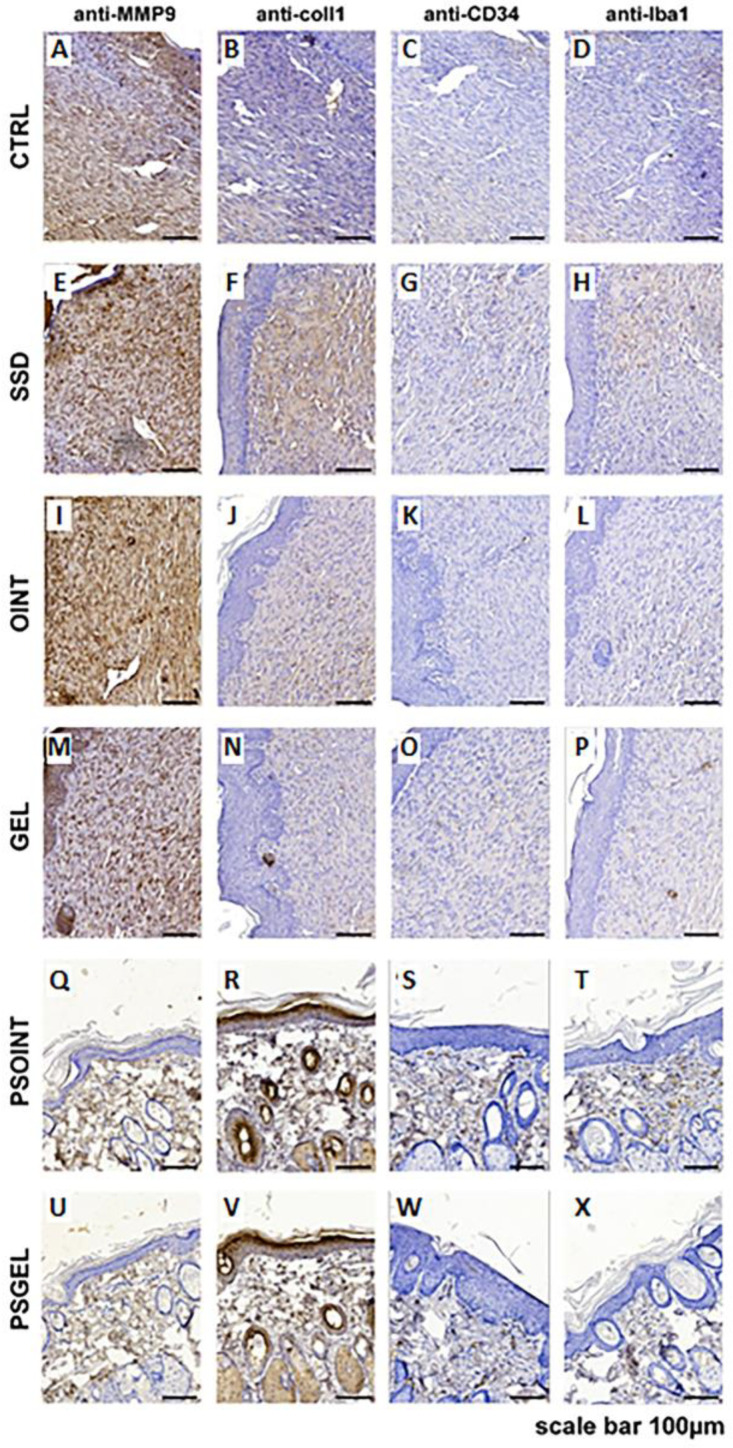
Representative immunohistochemical staining of MMP9, collagen I, CD34, and Iba1 in some animal groups. Scale bar, 100 μm. CTRL-untreated control group (**A**–**D**); SSD-1% silver sulfadiazine group (**E**–**H**); OINT-ointment base group (**I**–**L**); GEL-gel base group (**M**–**P**); PSOINT-*P. sibrica* essential oil ointment group (**Q**–**T**); PSGEL-*P. sibirica* essential oil gel group (**U**–**X**).

Histologic analysis of the control and all experimental groups indicates a complete re-epithelialization of the epidermal layer. In groups PSGEL and PSOINT, we noticed full epidermal thickness recovery, while in all other groups, especially in the region of resection, it was a little thinner. On the other side, cytological characteristics of the epidermis between the groups were preserved. However, the most noticeable changes could be observed in the superficial and primarily in the deep layers of the dermis. The most severe reparative fibrosis was observed in the CTRL group (Figure 7A) with a focal infiltration of neutrophil granulocytes. Moderate fibrosis, but still without restitution of adnexa, was observed in the SSD, OINT, and GEL groups, respectively (Figure 7B–D). Complete restitution of dermal adnexa, such as sebaceous and sweat glands and hair follicles without the presence of fibrosis, were observed in the group’s *Pinus sibirica* ointment base and gel base (Figure 7E,F). Using MMP9, we noticed predominantly faint staining of the cytoplasm of the fibroblast in the dermis of CTRL, SSD, OINT, and GEL groups (Figure 8A,E,I,M) while in PSOINT and PSGEL, according to restitution of epidermis and dermis, we did not identify an MMP9-activated fibroblast (Figure 8Q,U). Considering that 21 days passed after wounding, in SSD animal groups, we noticed the most pronounced production of collagen I (Figure 8F), while in CTRL, OINT, and GEL, it was lower (Figure 8B,J,N). The PSOINT and PSGEL group present a distribution of collagen 1, which we usually find in normal skin. Inside the fibrous scar, we identified CD34-positive capillary vessels. Looking at the vascularisation of the scar, there were no differences among groups (Figure 8C,G,K,O). The presence of capillary vessels in the PSOINT and PSGEL groups was arranged as usual (Figure 8S,W). The identification of Iba1 positive macrophages indicated that the maturation of fibrous tissue was not finished completely (Figure 8D,H,L,P) according to the PSOINT and PSGEL group, where presence was very rare (Figure 8T,X).

## 4. Discussion

The wound-healing process in diabetic patients is still a major challenge due to changed cellular and biochemical events, and the currently available treatment is not efficient and safe enough. Therefore, researchers are seeking novel strategies to achieve rapid healing activity, improve the quality of a patient’s life, and diminish the economic burden on the healthcare system [28]. Nowadays, natural compounds and products have been rediscovered by the medical profession and are gaining acceptance for treating many dermatological diseases involving the treatment of wounds. Since earlier scientific articles showed different effects of Siberian pine products [29,30], we assumed that PSEO incorporated into topical preparations (ointment or gel) could improve the wound-reparation process through oxidative stress reduction, increased collagen production, and changes in tissue structure. In this sense, our objective was to investigate the influence of essential oil-based formulations on the wound-healing profile under diabetic conditions and its side effects for dermal use.

At the beginning of our research, we determined the chemical compounds present in PSEO. The most dominant compounds were monoterpenes such as α-pinene, β-pinene, and δ-3-carene. Given the literature reports, our results of the chemical composition of Siberian pine essential oil are consistent with previous investigations with minor deviations. The quantity of chemical components of essential oil depends on various factors such as the part and health of the tree, needle maturity, season, and geographic location [14]. It has been known that monoterpenoids present the main ingredients of PSEO (68% from leaves and 78% from cones) [17]. On the other hand, the percentage of sesquiterpenoid components of the essential oil is 5–10% [31]. Concerning the geographic origin, investigations showed that PSEO from Mongolia is rich in δ-3-carene compared to essential oil from Kazakhstan [32,33]. Because some terpenes, as bioactive compounds of essential oils, can autoxidize and induce potential allergen reactions, it was inevitable to determine the safety properties of tested formulations. After incorporating *Pinus Sibirica* essential oil in ointment and gel, an acute dermal irritation test was performed. The Siberian pine ointment and gel did not exert any sign of adverse skin reaction, making them safe for topical treatment. Despite the widespread traditional usage of PSEO for different kinds of dermatological disorders, scientific evidence of the safety profile has not been reported until now. 

The rheological properties of topical preparations are very important because they reflect the physical stability of the semi-solid formulations during manufacturing, packaging, and even dispensing the product from the tube. Furthermore, testing the rheological profile of the topical product can reveal information about its ability to remain on the skin long enough to ensure efficacy as well as to affect the rate of active substance release from the preparation itself. Therefore, we investigated the rheological profile of the PSEO gel, PSEO ointment, and bases (gel and ointment) to predict product quality and its performance on the skin. Parameters describing the physical stability of prepared topical formulations, both gel and ointment, were desired. Observing flow curves, we noticed that the physical stability of products depends on the shear rate range. Similar values of shear stress were noticed in tested ointments and gels. Moreover, adding PSEO to topical preparations did not change flow behavior. A previous investigation revealed that topical administration of ointments and gels is easier due to the pseudoplastic behavior of semi-solid dosage forms. Actually, increased shear rates lead to an easier flow of semi-solid products, while decreased shear rates will induce higher product consistency, leading it to stay longer on the skin [34]. As we expected, shearing caused the breaking of the internal structure of both systems by decreasing the viscosity, which can enable better dermal administration. Given the above-mentioned results, we can conclude that the chosen bases for incorporating the *P. sibirica* essential oil have adequate properties (easy spreading and good adhesion) that are suitable for skin application [35].

After confirmation of the safety and stability of topical formulations, we focused on investigating the influence of *P. sibirica* essential oil preparations on the wound-healing process in diabetic rats by comparing them to commercially available agents and vehicles. Interestingly, both the ointment and gel with Siberian pine essential oil showed great wound-healing potential through two important phases: wound contraction and period of epithelialization of the wound area. Thus, the rate of wound closure is directly proportional to the efficacy of treatment. The enhanced contractile property caused by *P. sibirica* essential oil formulations is probably due to the presence of monoterpenes as the main ingredients of essential oil. Previous research showed accelerated wound-healing activity of α-pinene as the main constituent of numerous essential oils, which have the ability to penetrate into the skin and influence different phases of wound recovery [19]. In addition to the ability to modulate all phases of wound repair, essential oils promote tissue regeneration via enhanced collagen biosynthesis, wound contraction rate, and cellular proliferation, attributed to the synergistic effects of sesquiterpenes and monoterpenes [36]. Considering the fact that Siberian pine products can modulate inflammatory response, impact oxidative stress parameters or kill microorganisms [29,30], we may assume that the essential oil of *Pinus sibirica* promotes the healing process via diffusion through the skin. In the excision wound model, we noticed the highest wound closure and the shortest epithelization period in the PSEO ointment (98.14%) and PSEO gel (96.28%) groups in comparison to the control and vehicle groups. Our observations are in accordance with previously determined investigations with extracts rich in terpenoids [37]. 

Furthermore, collagen, as the most abundant extracellular protein, provides structural support to tissues and organs and plays a central role in the wound-healing process. In fact, various factors, such as diabetes, can influence collagen production, thus affecting the duration of the period of wound regeneration. In this sense, we aimed to measure collagen content in skin tissue through the evaluation of the level of hydroxyproline, an amino acid responsible for collagen synthesis and deposition in the tissue, thus indicating the stage of wound healing [27]. The outcomes were unequivocal, revealing a significant increase in hydroxyproline levels in wound tissue treated with *P. sibirica* formulations (ointment: 9.69 µg/mg dry tissue; gel: 7.26 µg/mg dry tissue) after three weeks of topical application. The most prominent level of hydroxyproline was observed in the group treated with the PSEO ointment. Based on these results, we can conclude that the topical application of the PSEO ointment and PSEO gel for three weeks contributed to an increased synthesis of collagen and a faster rate of tissue reparation in the excision type of diabetic wounds. In accordance with our results, an increased level of hydroxyproline was determined in tissues treated with ointments containing essential oils of some *Pinus* species (range: 17.54–30.60 µg/mg dry tissue) [38]. Moreover, the results of our study coincide with a study showing that α-pinene plays an important role in wound contraction by contributing to collagen formation and reducing inflammation [19]. The augmented hydroxyproline content and more efficient tissue reformation observed in groups treated with Siberian pine essential oil-based formulations were further substantiated through meticulous histopathological analyses. In brief, we determined a notable increase of collagen 1 positive fibrocyte presence and augmented density of collagen fibers after treatment with *P. sibirica* ointment and gel. Our examination supports the notion that topical formulations with Siberian pine essential oil enhance wound healing through its capacity to stimulate collagen synthesis, leading to more resilient and proficiently reestablished tissue. Moreover, apart from the evident increase in collagen deposition, another process that affects wound-healing activity is angiogenesis, which is additionally impaired by diabetes mellitus [39]. A key player in the intricate orchestration of wound re-epithelisation is the family of MMPs (matrix metalloproteinases), as they play a vital role in each phase of wound healing via regulating angiogenesis and managing the degradation and deposition of extracellular matrix. Our specific interest was MMP-9 since it is involved in the process of breaking down the extracellular matrix, promoting angiogenesis, and regulating pro-angiogenic cytokines. In our current investigation, the group of animals treated with PSEO ointment and PSEO gel exhibited a pronounced reduction in MMP-9 expression, underscoring the protective effects of our natural topical formulations. Our results are in accordance with previous studies, which proved that reduction in MMP-9 expression is a promising indicator of the effective wound-healing process [29]. Furthermore, we observed encouraging results in terms of the absence of CD34-positive structures and profound reduction in Iba1-positive macrophages after the application of the *Pinus sibirica* ointment and gel. These findings strongly correlate with the process of wound contraction, thus emphasizing that the Siberian pine essential oil may serve as a valid strategy in the management of diabetic wounds. Previously conducted investigations also suggested that a lack of positive CD34 structures is associated with the efficient healing of wounds [40].

Additionally, it is widely acknowledged that oxidative stress occurs when the body produces more pro-oxidants (reactive oxygen and nitrogen species) relative to antioxidants. Moreover, the regulation of oxidative stress plays an important role in the wound-repair process. Reduced production of reactive oxidative species (ROS) and/or enhanced antioxidant activity may provide faster wound repair and improve disturbed wound healing processes [41]. Therefore, we aimed to determine if plant-based topical formulations can modulate systemic oxidative status after three weeks of daily application. Our study showed higher production of mostly pro-oxidants such as H_2_O_2_, O_2_^−^ and TBARS in untreated and vehicle groups, which probably prolonged the wound-healing process. Earlier investigation confirmed that overproduction of ROS can cause cell damage and death through various mechanisms. On the other hand, attenuation of lipid peroxidation and elimination of pro-oxidants leads to the promotion of wound healing. We noticed that the *Pinus sibirica* ointment topical treatment showed a remarkable reduction of the above-mentioned pro-oxidants (2.23 nmol/mL, 1.23 nmol/mL, and 1.3 µmol/mL, respectively) as well as Pinus sibirica gel treatment (2.50 nmol/mL, 1.90 nmol/mL, and 2.00 µmol/mL, respectively) relative to controls (4.08 nmol/mL, 3.51 nmol/mL, and 3.27 µmol/mL, respectively). Furthermore, enhanced activity of SOD and CAT, as two important antioxidant enzymes, was observed after three weeks of treatment with both PSEO semi-solid formulations (ointment: 41.72 U/gHb × 10^3^ and 4.93 U/gHb × 10^3^, respectively; gel: 35.61 U/gHb × 10^3^ and 5.20 U/gHb × 10^3^, respectively), which lead to protection of cells in the wound area from oxidative damage, thus reducing the time for wound healing. This result was expected due to the already confirmed antioxidant properties of *P. sibirica* essential oil or terpenes [42,43].

Future research efforts should be directed toward conducting well-designed clinical trials to confirm the safety and efficacy of the *P. sibirica* essential oil formulation. Such validation will strengthen its clinical utility and facilitate its promising incorporation into evidence-based medical approaches. Moreover, future investigations can be directed to ensuring sufficient stability of topical semi-solid delivery forms (microcapsules, emulgels, emulsions, etc.) for Siberian pine essential oil by processes such as microencapsulation and high-pressure particle reduction [37].

## 5. Conclusions

- Conducted phytochemical analysis of PSEO revealed the presence of terpene compounds, which, in synergy, attributed to a significant wound-healing effect in animal models;
- Rheological measurements for the PSEO ointment and gel proved structure flexibility, indicating a good ability to spread on the skin;
- Novel plant formulations with *P. sibirica* essential oil are safe for application since there were no signs of dermal irritation;
- The PSEO ointment and gel formulations demonstrated a significant wound-healing effect in the diabetic rat model, supported by the increased contraction of wound area as well as hydroxyproline content, attenuation of oxidative stress, and histopathological analyzed markers.

## Figures and Tables

**Table 1:** Ointment base and *Pinus sibirica* essential oil ointment formulations.

Ingredient	Ointment Base (%)	Ointment PSEO (%)
Cholesterol	5.00	5
Lanolin	15.00	15
Parafinum liquidum	15.00	15
Vaselinum album	65.00	65
PSEO *	/	0.5

* PSEO, Pinus sibirica essential oil.

**Table 2:** Gel base and *Pinus sibirica* essential oil gel formulations.

Ingredient	Gel Base (%)	Gel PSEO * (%)
Carbomer 940	0.50	0.50
Propylene glycol	10.00	10.00
TEA 10%	q.s	q.s
Sodium benzoate	0.20	0.20
PSEO *	/	0.5
Aqua purificata	ad 100.00	ad 100.00

* PSEO-Pinus sibirica essential oil; TEA-triethanolamine.

**Table 3:** Chemical composition of *Pinus sibirica* essential oil.

Peak No	R.I. *	Compound	Rt (min)	%
1	934	α-Pinene	7.481	40.19
2	938	Camphene	7.907	1.17
3	974	β-Pinene	8.804	19.64
4	983	β-Myrcene	9.146	1.13
5	1007	δ-3-Carene	9.902	16.14
6	1011	α-Terpinen	10.102	0.22
7	1019	o-Cymene	10.386	2.06
8	1020	D-Limonene	10.553	9.92
9	1022	Eucalyptol	10.66	0.12
10	1079	Terpinolene	12.825	0.25
11	1272	Bornyl acetate	21.099	0.13
12	1403	Longifolene	26.112	3.18
13	1424	Caryophyllene	26.668	1.97
14	1452	Humulene	28.028	2.59
15	1512	δ–cadinene	30.734	0.27
16	1578	Caryophyllene oxide	33.047	0.34
Total % of identified compounds				99.32

* R.I.-Retention indexes determined relative to a series of n-alkanes (C9–C24) on a HP 5 MS column.

## Data Availability

The collected data in this study are available from the corresponding author upon reasonable request.
